# Suppression of Inflamm-Aging by *Moringa oleifera* and *Zingiber officinale* Roscoe in the Prevention of Degenerative Diseases: A Review of Current Evidence

**DOI:** 10.3390/molecules28155867

**Published:** 2023-08-03

**Authors:** Nur Fatin Nabilah Mohd Sahardi, Suzana Makpol

**Affiliations:** Department of Biochemistry, Faculty of Medicine, Universiti Kebangsaan Malaysia, Jalan Yaacob Latif, Bandar Tun Razak, Cheras, Kuala Lumpur 56000, Malaysia

**Keywords:** anti-inflammatory agents, degenerative disease, *Moringa oleifera* Lam, inflamm-aging and *Zingiber officinale* Roscoe

## Abstract

Inflammation or inflamm-aging is a chronic low-grade inflammation that contributes to numerous types of degenerative diseases among the elderly and might be impeded by introducing an anti-inflammatory agent like *Moringa oleifera* Lam (moringa) and *Zingiber officinale* Roscoe (ginger). Therefore, this paper aims to review the role of moringa and ginger in suppressing inflamm-aging to prevent degenerative diseases. Various peer-reviewed publications were searched and downloaded using the reputed search engine “Pubmed” and “Google Scholar”. These materials were reviewed and tabulated. A comparison between these previous findings was made based on the mechanism of action of moringa and ginger against degenerative diseases, focusing on their anti-inflammatory properties. Many studies have reported the efficacy of moringa and ginger in type 2 diabetes mellitus, neurodegenerative disease, cardiovascular disease, cancer, and kidney disease by reducing inflammatory cytokines activities, mainly of TNF-α and IL-6. They also enhanced the activity of antioxidant enzymes, including catalase, glutathione, and superoxide dismutase. The anti-inflammatory activities can be seen by inhibiting NF-κβ activity. Thus, the anti-inflammatory potential of moringa and ginger in various types of degenerative diseases due to inflamm-aging has been shown in many recent types of research.

## 1. Introduction

Inflammation is a normal body response towards cellular injury or trauma that serves as a mechanism for eliminating noxious agents and damaged tissue. It also initiates tissue repair processes to maintain normal homeostasis in the body [1]. Inflammation related to the aging process is known as “inflamm-aging”. Inflamm-aging or chronic low-grade inflammation occurs concomitantly with the advancing of age. A previous study showed that inflamm-aging was associated with the accumulation of cytokines such as tumour necrosis factor alpha (TNF-α), interleukin 6 (IL-6), interleukin 1 alpha (IL-1α), and C-reactive protein (CRP) [2]. During inflammation, macrophages and monocytes are activated and release TNF-α and IL-6, which are essential to initiate the progression of systemic processes.

The inflammatory response can be categorised into two types, which are the acute inflammatory response and the chronic inflammatory response. An acute inflammatory response is one of the body’s innate defence mechanisms against infectious or non-infectious agents [3]. It occurs immediately but is non-specific. However, a chronic inflammatory reaction occurs when a critical inflammatory mechanism fails to remove the tissue injury or the pathogen penetrating the body [4]. Prolonged and persistent exposure to chronic inflammation could contribute to the progression of chronic conditions and serious adverse health outcomes, particularly cardiovascular disease, type 2 diabetes mellitus, Alzheimer’s disease, sarcopenia, and osteoarthritis [4]. The topic and scope of this paper were identified before the information collection process, followed by construction of the title of the manuscript. Then, all the previous studies reporting the subject matter within the scope of the review, including peer-reviewed publications, books, and monographs, were downloaded and retrieved from the reputable search engines “Pubmed” and “Google Scholar” using specific search terms or keywords. The previous research information was extracted and analysed before synthesising them into tables and figures. The results were summarised, and conclusions from this study were drawn based on the tabulated table.

## 2. Causes of Inflamm-Aging

Inflamm-aging can be triggered by several factors related to the changes during aging, such as an alteration in the redox balance, a decrease in effective autophagy, and an increment in senescence-associated secretory phenotype (SASP) [5]. An alteration in the cellular redox balance is associated with a higher level of oxidative stress. The body’s imbalance between the reactive oxygen species (ROS) level and antioxidant defence is known as oxidative stress [6]. ROS are standard side products of cellular metabolism. Excessive production of ROS can react with macromolecules such as nucleic acids, lipids, and proteins and later could contribute to DNA damage and cell membrane damage. ROS are produced from several origins, such as the mitochondria, the nicotinamide adenine dinucleotide phosphate (NADPH) pathway, and the cyclooxygenase (COX) pathway [7]. ROS are released through the mitochondrial electron transport chain in the mitochondria, which consist of complexes I, II, and III. As age advances, mitochondria become sluggish and produce less energy, resulting in mitochondrial dysfunction. ROS can also be produced from the NADPH pathway, which is essential for host defence and bacterial killing [8]. This occurs when phagocytes detect the presence of endogenous or exogenous danger in the body before it fuses with the plasma membrane to form a phagosome. The phagosome will undergo a phagocytic burst and release ROS.

Moreover, the COX pathway is vital in converting lipids using the cyclooxygenase-2 (COX-2) enzyme into prostaglandin H2 (PGH2) and releasing superoxides [9]. The accumulation of superoxides could result in the elevation of oxidative stress. Excessive ROS production triggers inflammasome formation before stimulating the nuclear factor-κβ (NF-κβ) pathway. Then, the stimulated NF-κβ initiates the inflammatory mediators, particularly IL-6, interleukin 1 beta (IL-1B), TNF-α, interleukin 2 (IL-2), and chemokines, which later cause an inflammatory response in the body.

Inflamm-aging is also associated with the poor functioning of autophagy. Autophagy is a cell mechanism that removes damaged proteins and large aggregates that undergo apoptosis or programmed cell death. Two major pathways are responsible for eliminating this cellular protein: the ubiquitin–proteasome system and the lysosome–autophagy system [10]. With increasing age, this mechanism is disturbed, accumulating damaged material in the body and reducing cellular efficacy. Sarcopenia is one of the diseases associated with autophagy dysregulation [11]. In sarcopenia, this dysregulation causes an accumulation of interferences with normal myofiber function, which contributes to the imbalance between protein production and degradation. Excessive autophagy in the muscle can cause protein degradation, resulting in cellular stress and loss of skeletal muscle mass [12]. However, insufficient autophagy in muscle contributes to the abnormal aggregation of misfolded proteins.

The high production of senescent cells also triggers inflamm-aging during aging. The two significant signs of cellular senescence are the generation of SASP, which causes the irreversible arrest of cell proliferation. Cellular senescence is a response to stress initiation via telomere attrition, genomic instability, protein misfolding, ROS production, and DNA damage, which are increased in older people [13]. During aging, the rate of SASP generation is higher than its clearance rate. Inflammation due to the presence of SASP contributes to several degenerative diseases, including atherosclerosis, cancer, and diabetes [14,15,16].

## 3. Mechanism of Inflammation

**Figure 1:**
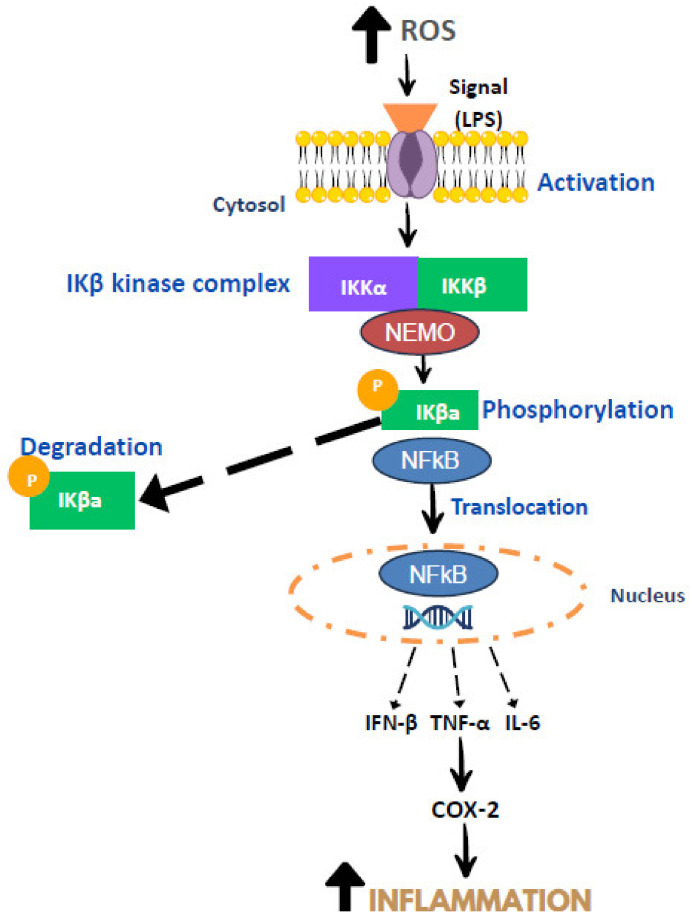
Mechanism of inflammation. Adapted from [17,18,19,20].

InflInflammation which is associated with high production of ROS, is initiated by tissue injury and infection, which later activate mast cells and tissue macrophages, contributing to the generation of inflammatory mediators, particularly vasoactive amines, chemokines, cytokines, and products of proteolytic cascades [17]. Lipopolysaccharides (LPS) function as cell signals which later activate the NFκB pathway [18] (Figure 1). The activation of the NF-κB pathway involves IkappaB kinase (IκB kinase) or IKK, which comprises IKKα and IKKβ [19]. Upon activation of NF-κβ by various stimuli, the IKK will interact with inhibitory IKβa and cause phosphorylation and degradation of IKβa. The phosphorylation and degradation of IKβa contributes to the displacement of NF-κB into the nucleus. Subsequently, activation of the target gene will occur, producing pro-inflammatory cytokines such as interferon-beta (IFN-β), IL-1, TNF-α, and IL-6. After that, COX-2 is released, and this COX-2 produces prostaglandin that promotes inflammation [20]. Continuous inflammation will trigger various degenerative diseases, including diabetes, cardiovascular disease, hypertension, etc.

These degenerative diseases can be prevented by introducing medicinal herbs or plants. Previous studies have shown that numerous medicinal herbs can be used as anti-inflammatory agents in preventing degenerative disease, including ginger, *Moringa oleifera*, *Centella asiatica*, palm oil, and others. Moringa and ginger have been consumed as a natural remedy from ancient times. Both herb plants are rich with numerous antioxidants that effectively work against multiple diseases and ailments. Therefore, this review will emphasize the effect of *Moringa oleifera* and ginger consumption as anti-inflammatory agents in preventing some degenerative diseases.

## 4. *Moringa oleifera* and *Zingiber officinale* Roscoe

**Figure 2:**
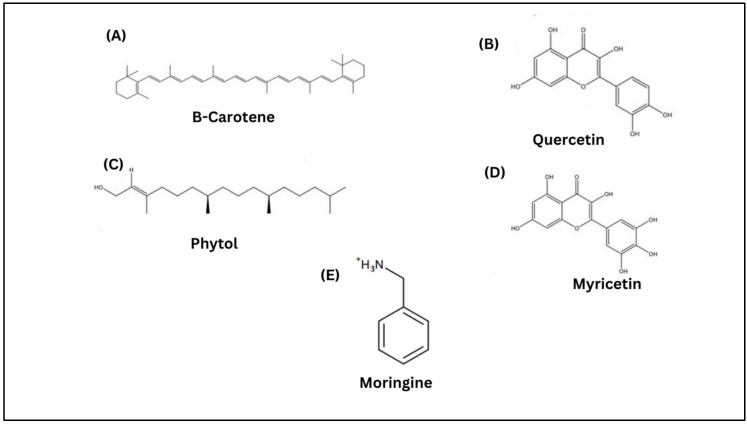
Chemical structures of bioactive components of *Moringa oleifera:* (**A**) beta carotene, (**B**) quercetin, (**C**) phytol, (**D**) myricetin, and (**E**) moringine.

Moringa (*Moringa oleifera* Lam) (MO) comes from the Moringaceae family, and the Moringa genus is among the Indian traditional herbs used widely for health benefits. This plant is also known as “the magic tree” among the local people, which is related to its ability to treat various types of diseases [21]. MO is also recognised as Mulangay, Horseradish tree, Benzolive, Mlonge, Drumstick tree, Saijihan, Sajna, Kelor, and Marango [22]. MO seed, root, leaves, and flowers are useful for medicinal purposes. MO is widely distributed in North West India, Africa, the Caribbean, South East Asia, and South America [21]. A previous study found that the MO plant has been used extensively in treating skin infections, diabetes, anaemia, hypertension, and other types of diseases. MO is rich in bioactive components, particularly beta-carotene, quercetin, phytol, myricetin, and moringine [23]. These bioactive compounds have contributed to several biological events through, for example, their antioxidant, anti-inflammatory, hepatoprotective, antidiabetic, antiproliferative and cardioprotective activities [24,25,26,27]. Chemical structures of the bioactive components of moringa are displayed in Figure 2.

**Figure 3:**
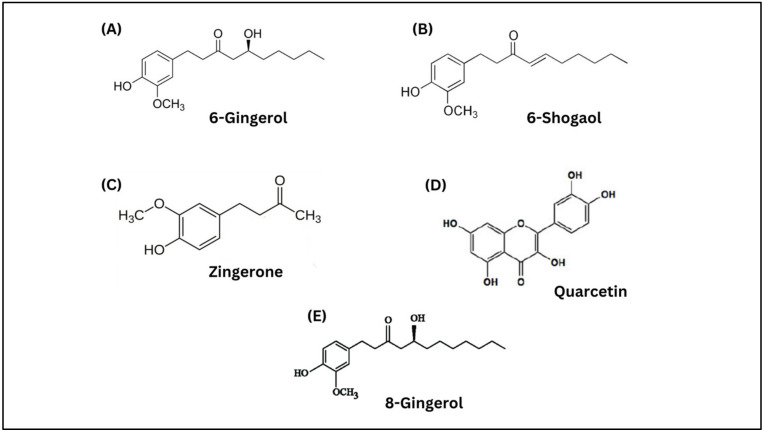
Chemical structures of bioactive components of ginger extract: (**A**) 6-gingerol, (**B**) 6-shogaol, (**C**) zingerone, (**D**) quercetin, and (**E**) 8-gingerol.

*Zingiber officinale* Roscoe (ginger) is another medicinal herb studied as an anti-inflammatory agent in the pathogenesis of some degenerative diseases caused by inflammation processes [28]. Ginger, from the Zingiberaceae family and the *Zingiber* genus, is used as a spice or flavour in cooking. It is also used as a medicinal herb for treating numerous diseases, including hypertension, diabetes, migraine, nausea, cardiovascular disease and Alzheimer’s disease [29,30,31,32]. Ginger originated in tropical and subtropical Asia, China, Far East Asia, India, and Africa [33]. Ginger is composed of various types of bioactive components, including 6-gingerol, 6-shogaol, 8-gingerol, zingerone, and quercetin (Figure 3), which exhibit not only an anti-inflammatory effect but also antibacterial, anticancer, antidiabetic, gastro-protective, antioxidant, and neuroprotective effects [34,35,36]. A summary of the comparison between MO and ginger is shown in Table 1.

## 5. The Anti-Inflammatory Properties of *Moringa oleifera* and Ginger

In a previous study, several active compounds, particularly phenols, alkaloids, flavonoids, tannins, β-sitostenone, vanillin, β-sitosterol, moringine, moringinine and hydroxymellein, have been found to promote the anti-inflammatory property of MO [37]. β-Sitosterol, for example, can downregulate inflammatory cytokines, such as IL-6, interleukin 8 (IL-8) and TNF-α, together with induction of haem oxygenase-1 (HO-1), an anti-inflammatory protein [37]. In addition, the inhibition of NLRP3 inflammasomes and the production of active forms of pro-IL-1β and caspase-1 were demonstrated. In another study, quercetin reduced inflammation by inhibiting the action of NF-κβ and subsequent NF-κβ-dependent downstream events of inflammation [38]. This was supported by another study that found that MO significantly repressed the NF-κβ signaling pathway through upregulation of the inhibitor of κβ expression together with the downregulation of pro-inflammatory mediators [39]. Meanwhile, the active compound in leaves of MO significantly suppressed protein expression of inducible nitric oxide synthase (iNOS) β, COX-2 and nitric oxide (NO) and inflammatory markers induced by LPS, particularly prostaglandin E_2_ (PGE), IL-1β, TNF-α and IL-6 [40]. However, it increased the expression of interleukin 10 (IL-10) by inhibiting the signaling cascades activating NF-κβ. Tan et al. [41] demonstrated that the MO flower possesses the active compounds quercetin and kaempferol, which significantly reduce the generation of NO and downregulate the existence of pro-inflammatory (IL-1β, TNF-α, PGE2 and IL-6) and inflammatory (NF-κβ, COX-2 and iNOS) cytokines. However, this flower extract enhances the expression of IL-10 and the nuclear factor of kappa light polypeptide gene enhancer in B-cells inhibitor, alpha (IκB-α), which are anti-inflammatory cytokines. The anti-inflammatory property of the flower extract was shown by preventing NF-κβ activation and translocation into the nucleus before inhibiting the production of various inflammatory proteins.

**Figure 4:**
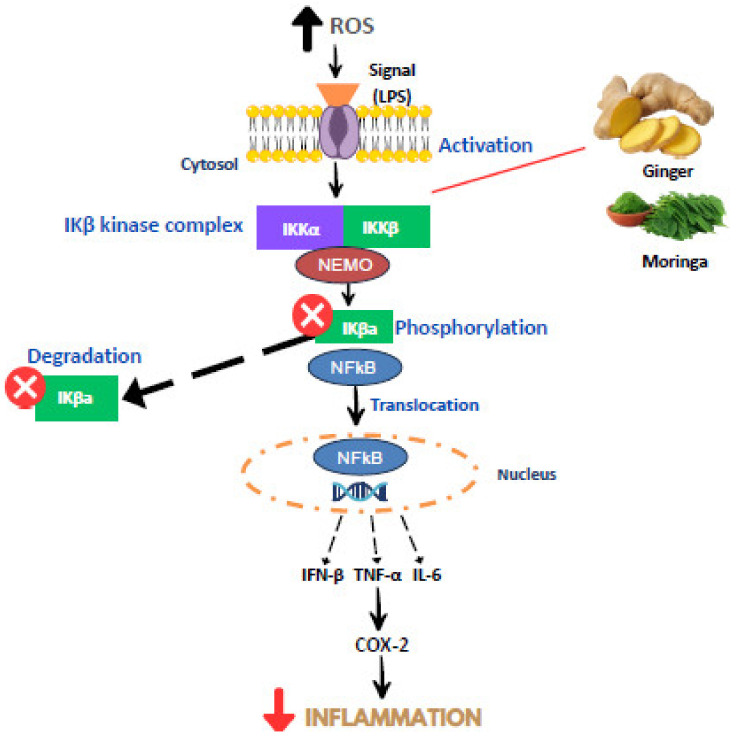
Mechanism of moringa and ginger in preventing inflammation. Adapted from [38,42].

Meanwhile, for ginger, 6-shogaol, 6-paradol, and 1-dehydro-6-gingerol possess the most potent anti-inflammatory activity [42]. These active compounds significantly reduce TNF-α and trigger a decrement in the pro-inflammatory cytokines IL-6 and IL1-β [42]. Ginger has been proven to prevent the stimulation of neutrophils and macrophages and affect the migration of monocytes and leukocytes [42]. In another study, it was shown that active compounds such as phenolic acid and flavonoid acid reduced IL-10 levels; at the same time, they increased the expression of pro-inflammatory cytokines, particularly IL-1, IL-6, TNF-α, and interferon-gamma (IFNγ) [43]. The ginger extract has also been proven to increase inflammatory cytokines by impeding NF-κβ and decreasing PGE2, nitrite, and interleukin 8 (IL-8) levels [44]. This was in line with another study that demonstrated that gingerol and shogaol in ginger lowered the expression of the hepatic inflammation markers TNF-α and IL-6 via suppression of the NF-κβ activity [45]. Figure 4 shows the potential mechanism of ginger and MO in suppressing inflammation pathways.

## 6. The Role of MO and Ginger in Preventing Age-Related Degenerative Diseases

Since many age-related degenerative diseases are caused by inflammation, the function of MO and ginger as anti-inflammatory agents has been studied in vivo, in vitro, and in human studies. Numerous researchers have recognised the use of these medicinal herbs as anti-inflammatory agents in preventing degenerative diseases, especially neurodegenerative disease, cardiovascular disease, diabetes, and cancer.

### 6.1. Effect of MO and Ginger on Type 2 Diabetes Mellitus

The anti-inflammatory activities of MO have been seen to prevent type 2 diabetes mellitus [46]. The administration of a methanolic extract of MO to Wistar rats with nephrotoxicity induced by diabetes restored the typical unfavourable side effects caused by streptozotocin (STZ) by lessening the presence of TNF-α and IL-6 [47]. Furthermore, this extract significantly reduced malondialdehyde (MDA) levels and glutathione peroxidase (GPx) activity and increased catalase (CAT) activity, suggesting MO’s capability as an antioxidant agent. This was similar to the previous study by Kamaliani et al. [48]. They found that the aqueous leaf decoction of MO led to an upregulation of the mRNA expression of neurogenin 3 (Ngn3), vascular endothelial growth factor (VEGF), insulin like growth factor-1 (IGF-1), insulin promoter factor 1 (PDX-1), and glucose transporter 1 (GLUT-1) in diabetic rats. While fasting, blood glucose (FBG) and MDA levels were reduced after MO treatment. The histopathology of the liver, kidney, and pancreatic tissues of diabetic rats also improved with MO treatment. In another finding, MO leaves could reduce blood glucose, insulin, and TNF-α levels as well as follicle counts in a polycystic ovary syndrome (PCOS) diabetic mice model [49]. Moreover, in a study involving a high-fat-diet mouse model, moringa isothiocyanate-rich seed extract (MIC-1) reduced body weight, improved glucose tolerance, reduced inflammatory gene expression, decreased adiposity, and increased antioxidant gene expression [50]. These positive effects of MO were shown to occur through the activation of Nrf2, which is driven by MIC-1. In addition, they demonstrated that this MO extract inhibited gut microbiota, probably by reducing metabolic endotoxemia and enhancing metabolic health. Meanwhile, in a previous in silico study, Huang et al. [51] showed that the major bioactive compounds that improved insulin resistance in diabetes were glycosidic isothiocyanates and glycosidic benzylamines. These two types of bioactive compounds are involved in insulin related pathways and inflammatory responses by acting on tyrosine-protein phosphatase non-receptor type 1 (PTPN1), the proto-oncogene tyrosine-protein kinase (SRC), and caspase-3 (CASP3). MO extract treatment increased glucose uptake and modulated the expression of SRC and PTPN1.

Various studies have shown that ginger can be an anti-inflammatory agent in treating type 2 diabetes mellitus [52,53]. Carvalho et al. [54] found that the daily consumption of ginger in a 3-month human study reduced the level of triglycerides, fasting blood sugar, and LDL-cholesterol in patients with type 2 diabetes mellitus. Meanwhile, in an animal study, ginger extract ameliorated the hyperlipidaemia, hyperglycaemia, and kidney function of diabetic rats [55]. The histological alteration in the kidney due to diabetes was also minimised by ginger extract. After treatment with ginger extract, the levels of IL-6 and TNF-α were reduced, while the levels of cytochrome c and caspase-3 were increased. The increment in cytochrome C and caspase 3 indicated the ability of ginger extract to prevent apoptosis in the kidney of diabetic rats. In another animal study, it was found that treatment with ginger extract in a diabetic rat model led to the reduction in hippocampal and cortical MDA levels and improved catalase (CAT) activity [56]. It also increased the level of superoxide dismutase (SOD) and total thiols in cortical and hippocampal tissues, contributing to memory improvement in STZ-induced diabetes rats. A previous study also reported that ginger treatment in diabetic rats could improve glucose tolerance, insulin content, and gene expression of Claudin 3 and SOD1 [57]. It also reduced the expression of genes involved in mitophagy (LC3B, PINK1 and LC3B), biogenesis (TFAM and PGC-1α), mitochondrial fission (MFN1), fission (F1S1), and inflammation (NF-κβ).

### 6.2. Effect of MO and Ginger on Cardiovascular Disease

Current findings report that MO treatment positively affects cardiovascular disease. The administration of MO seed in spontaneously hypertensive rats displayed a protective effect against hypertension [58]. MO extract was reported to improve diastolic cardiac function by stimulating the peroxisome proliferator-activated receptor (PPAR)-α and -δ and plasmatic prostacyclin with decreased fibrosis in the left ventricle as well as reduced inter-septal thickness on the diastole relative wall thickness. It also lowered the level of triglycerides in the heart. Aju et al. [59] reported that the antioxidant potential of MO against oxidative stress in the heart was exhibited through the improved levels of plasma insulin, SOD, CAT, GPx, and glutathione reductase (GRD). MO treatment also led to the reduction in GSH, serum glucose, thiobarbituric acid reactive substances (TBARS), glycated haemoglobin, conjugated dienes (CD), and hydroperoxides (HP). A study by Randriamboavonjy et al. [60] demonstrated that consuming MO with food for 20 weeks reduced the circulating CRP and nitrites associated with the decrement in NF-κβ protein and iNOS expression. In addition, there was an upregulation in the expression of SOD and a reduction in the levels of circulating free 8-isoprostane, p47^phox^, and p22^phox^. Meanwhile, Saka et al. reported that MO seed oil significantly reduced cardiac lactate dehydrogenase (LDH), creatinine kinase (CK), and troponin levels, which induced cardiac injury in rats. Moreover, oral administration of this extract contributed to decreased cardiac levels of MDA and increased cardiac activities of SOD and GPx. The cardiac histomorphology also significantly improved with MO oil seed. In another study, MO seed improved aging-related endothelial dysfunction in Wistar rats [61]. The vascular protective effect of MO seed could be observed by the improved carbachol-induced relaxation in both mesenteric arteries and aortas. The improvement in the mesenteric arteries in terms of endothelial-dependent relaxation was associated with the endothelium-derived hyperpolarizing factor (EDHF)- dependent mechanism, while the improvement in the aorta was related to the activation of endothelial NO synthase, an increment in Akt signalling, and the downregulation of arginase-1.

Previous research also found that ginger’s anti-inflammatory properties protect against cardiovascular disease. A previous in vitro study demonstrated that ginger extract improved cardiovascular disease by inducing the relaxation of coronary arteries [62]. The ginger extract also increased vasoprotection by suppressing nitric oxide synthase and the COX pathway. These findings agreed with another in vitro study, which found that fresh ginger improved adenosine deaminase levels, ADP hydrolysis, and acetylcholinesterase activity in the lymphocytes of hypertensive rats [43]. In addition to the decline in interleukin 10 (IL-10), a concomitant increase in IL-6, IL-1, interferon-ϒ, TNF-α and serum butyrylcholinesterase activity and pro-inflammatory cytokine levels was seen in hypertensive rats. Additionally, ginger extract ameliorated ethanol-induced heart abnormalities in male Wistar rats. After six months of ginger treatment, there were significant decreases in the amount of 8-OHdG, β-myosin heavy chain (β-MHC) gene expression, and NADPH oxidase level [63]. However, the level of the paraoxonase enzyme increased significantly with ginger treatment.

### 6.3. Effect of MO and Ginger on Neurodegenerative Disease

Many findings have revealed the capability of MO to reduce inflammation in neurodegeneration-related diseases, especially Alzheimer’s disease (AD) and Parkinson’s disease. In AD, an animal study has shown that MO can be used as a prevention or treatment against oxidative stress and cognitive impairment [64]. In that study, MO extract was reported to reduce calpain activity, Hyc-induced tau hyperphosphorylation, and amyloid β (Aβ) production [64]. It restored the expression of some synaptic proteins (synaptophysin, PSD93, synapsin 1, and PSD95), which were reduced in Alzheimer-induced rats. The effect of MO on PD in in vitro studies can be seen through the activity of their active compound, isothiocyanate [65]. This active compound modulates the apoptotic and inflammatory pathways and oxidative stress in RAW 264.7 macrophages and a mouse PD model. For the inflammatory pathway, there was a reduction in the manifestation of TNF-α toll-like receptor 4 (TLR4) and IL-1β. In another in vitro study, the neuroprotective effect of MO leaf powder extract could be observed through antioxidative and mitochondrial regulation [66]. The MO extract increased the cell viability and reduced free radicals in a hydrogen peroxide (H_2_O_2_)-induced oxidative stress model in human neuroblastoma cells. It also improved the glutathione level and antioxidant enzyme activity and reduced the lipid peroxidation due to H_2_O_2._ MO extract inhibited mitochondrial dysfunction by regulating the calcium level and incrementing the mitochondrial membrane potential. The 70% ethanolic extract of MO seeds also displayed a neuroprotective property in a mouse model of cognitive impairment induced by scopolamine [67]. Treatment with MO seed led to an improvement in cholinergic reactivity and neurogenesis in the scopolamine-induced group. These changes were triggered by the enhancement of the cholinergic system and hippocampal neurogenesis by the Akt/ERK1/2/CREB signalling pathway. In a study by Onasanwo et al. [68], an MO-supplemented diet protected against the cortico-hippocampal neuronal degeneration associated with a scopolamine-induced spatial memory deficit in mice. The MO extracts reduced the levels of oxide-inflammatory stress markers such as MDA, nitrite, SOD and TNF-α, and restored cholinergic transmission (AChE) by inhibiting acetylcholinesterase and maintaining the neuronal integrity in mice brains.

Ginger also works effectively in neurodegenerative diseases. A previous study found that ginger extract contributed to the upregulation of brain-derived neurotrophic factor (BDNF) in an amnesia mouse model, which was triggered by the activation of protein kinase B/Akt as well as the cAMP-response element binding protein (CREB) signalling pathway [69]. Furthermore, Na et al. [70] found that ginger extract, especially 6-shogaol, showed both in vitro and in vivo neuroprotective effects in treating AD. Their in vitro study found that treatment with 6-shogaol inhibited the Aβ aggregation in HT22 cells, while the in vivo study demonstrated that 6-shogaol repressed the aggregation of Aβ in the animal brain, which indicates the potential of ginger in AD treatment. Another study supporting this found that ginger extract lessened memory impairment by inhibiting the neuronal cell loss and synaptic disruption caused by Aβ plaque aggregation [71]. In addition, 6-shogaol has been demonstrated to downregulate cysteinyl leukotriene 1 receptor (CysLT1R), a major factor in AD pathogenesis [72]. This downregulation contributed to the inhibition of CysLT1R/cathepsin B as well as reduced the Aβ deposition in the brain and ameliorated the behavioural deficits of the AD mice model.

### 6.4. Effect of MO and Ginger on Cancer

*Moringa oleifera* also works effectively as an anti-inflammatory agent in preventing cancer disease. Xie et al. [73] found that 4-[(α-L-Rhamnosyloxy)-benzyl] isothiocyanate (MIC-1) was an active substance in MO with anti-cancer activity. In an in vitro study, this active substance significantly inhibited the growth of five types of renal cell carcinomas, namely OSRC-2, 769-P, 786-O, SK-NEP-1, and ACHN cells. In 786-O and 769-P cells, MIC-1 inhibited cell migration and invasion, reduced the expression of matrix metalloproteinase (MMP)-9 and MMP-2, induced cell-cycle arrest and apoptosis, increased the Bax/Bcl-2 ratio, and decreased protein expression. Meanwhile, an in vivo study found that MIC-1 suppressed the growth of xenograft tumours and enhanced the Bax/Bcl-2 ratio in tumour tissues in mice. In another study, Siddiqui et al. [74] found that MO extract, particularly from the fruit part, promoted anti-proliferative activity against human hepatocellular carcinoma HepG2 cells by activating the caspase 3 enzyme and ROS-mediated apoptosis. In molecular docking analyses, they reported that active substances in MO fruit displayed drug-like candidates without any toxicity effect. The ability of MO leaves as an anti-cancer agent has also been demonstrated in a colitis-associated colon carcinogenesis model [75]. There was a decrease in lipid peroxidation and myeloperoxidase activity after treatment with MO. Pro-inflammatory cytokines in the serum, particularly IL-6, TNF-α, and IL-2, were significantly reduced in the colitis-associated colorectal cancer model. In human prostate PC-3 cancer cells, MO methanolic leaf extract promoted G0/G1 cell-cycle progression and apoptosis by downregulating the Hedgehog signaling pathway [76]. The downregulation of the Hedgehog signaling pathway could be observed via decreased mRNA expression of the GLI1 transcription factor and SMO protein. The findings by Mohd Fisall et al. [77] demonstrated that MO leaf extract induced early apoptosis and increased the expression of pro-apoptotic proteins, including Bax, caspase 8, and p53, in breast cancer cells.

Various studies have shown that ginger exhibits anti-inflammatory characteristics in treating cancer. A previous study reported that 6-gingerol repressed cell proliferation, enhanced the sub-G1 phase ratio, and depolarised the mitochondrial membrane potential of a human bladder cell line [78]. This active compound triggered cell death by downregulating B-cell lymphoma 2 (BCL-2) and survivin and upregulating the Bcl-2 associated X protein (Bax). The anticancer activity of 6-gingerol in this study was also demonstrated by the activation and regulation of caspase-3, caspase-9, and MAPKs. In another finding involving in vitro and in vivo studies, 6-shogaol was demonstrated to suppress cell migration and proliferation, which later contributed to cell-cycle arrest in the G_2_/M phase in cervical carcinoma cells [79]. Furthermore, 6-shogaol induced apoptosis via the mitochondrial pathway by downregulating the expression of p-Akt, p-mTOR, and Pl3K. Meanwhile, another in vivo finding demonstrated that 6-shogaol significantly inhibited cell proliferation and tumour growth in tumour tissues. Woźniak et al. [80] reported that 6-shogaol was able to improve the anticancer effect of chemotherapeutic agents, particularly 5-fluorouracil, oxaliplatin, and irinotecan, by increasing autophagy and apoptosis in colon cancer cells. In another study involving a mouse colorectal adenoma model, 6-shogaol treatment reversed the effect of Azoxymethane (AOM) and dextran sulphate sodium (DSS) in animal models by reducing colon weight, colon length, and the levels of NO, myeloperoxidase (MPO), H_2_O_2_, and TNF-α [81]. In contrast, levels of antioxidant enzymes such as SOD, CAT, glutathione (GSH), and glutathione S transferase significantly increased in the treatment group with 6-shogaol.

### 6.5. Effect of MO and Ginger on Kidney Disease

Many studies have reported MO as an anti-inflammatory agent in treating kidney disease. A study by Omodanisi, Aboua, and Oguntibeju [47] showed that MO displayed a high antioxidant capacity and improved the profile of serum biochemical markers, particularly SOD, CAT, GPx, GSH, and IL-6. Bioactive compounds in MO also contributed to a reduction in lipid peroxidation, decreasing MDA levels. Another finding supporting this study reported that MO could counteract the effect of the hepato-renal dysfunction induced by methotrexate and oxidative stress in mice [82]. They discovered that MO extracts reduced the amount of alanine aminotransferase (ALT), CAT, aspartate aminotransferase (AST), and SOD. MO also inhibits apoptosis by increasing the expression of XIAP (anti-apoptotic gene) and Bcl-2 and decreasing caspase 3 and Bax levels. Another study involving rats with acute kidney injury induced by glycerol showed that MO inhibited the expression of markers for inflammation, oxidative stress, and renal injury by modifying kidney injury molecule 1 (KIM-1) and NF-κβ signaling pathways [83]. The administration of MO leaf extract contributed to the reduced expression of IL-6, IL-1β, TNF-α, COX-2, and iNOS in renal tissue of type 2 diabetic mice [84]. The histopathological damage was diminished. In renal ischemia–reperfusion injury, MO extract has been shown to reduce the levels of advanced oxidation protein products (AOPP), MDA, protein carbonyl, creatinine and serum blood urea nitrogen (BUN) [85]. It also increased the activities of GPx and glutathione S-transferase (GST) while reducing the level of H_2_O_2_ and NO in renal tissue.

Ginger extract also exhibited the role of an anti-inflammatory agent in treating kidney-related diseases in an animal model. In a study using diabetic nephropathic rats, ginger alleviated the effect of hyperglycaemia induced by oxidative stress, apoptosis, and inflammation [55]. The levels of MDA, protein carbonyl, pro-inflammatory cytokines, caspase 3, and cytochrome c were reduced after treatment with ginger extract for six weeks. The ginger extract also minimised alterations in the diabetic rats’ kidneys. This was in line with another finding showing that ginger extract significantly ameliorated the alteration in kidney structure with the restoration of biochemical changes [86]. Research conducted by Rehman et al. [87] demonstrated that zingerone, a bioactive compound in ginger extract, reduced the level of inflammatory molecules, including TNF-α, IL-6, and IL-1β by delaying the stimulation of NF-κβ in streptozotocin/high-fat diet (STZ/HFD)-induced type 2 diabetic rats. The renal function of STZ/HFD rats also improved significantly, as shown by decreased KIM-1, creatinine, and BUN levels and suppressed expression of transforming growth factor beta (TGF-β) and lactate dehydrogenase (LDH). The anti-inflammatory effect of ginger extract was also observed in a study by Gabr et al. [88]. They found that renal toxicity induced by cadmium in rats decreased with ginger extract treatment. In this study, ginger extract was found to restore the total antioxidant content (TAC), renal function, histological changes, and maintenance of molecular DNA. This restoration was due to ginger’s free-radical scavenging and regenerative activities. This was reinforced by other research showing that treatment with ginger extract provides kidney protection against acute mercuric chloride intoxication [89].

Thus, MO and ginger can be utilised as substitute therapy to prevent degenerative diseases. Table 2 shows the current research findings (from in vitro, in vivo, and human studies) on the effects of MO in treating degenerative diseases. In contrast, the outcome of ginger treatment on degenerative diseases is shown in Table 3.

## 7. Conclusions and Perspective

In this paper, we reviewed the present evidence on the capabilities of MO and ginger in suppressing inflamm-aging to prevent degenerative diseases. Previous studies have revealed that prolonged exposure to inflammation, also known as inflamm-aging, could result in the pathogenesis of numerous degenerative diseases. Medicinal herbs like ginger and MO could prevent these degenerative diseases. The potential to prevent degenerative disease is associated with their active component activity. Many studies have shown that MO and ginger can reduce the level of inflammatory cytokines, including IL-6, TNF-α, IL-1, and MDA, indicating their potential to suppress inflamm-aging, thus proving that these medicinal herbs can reduce inflammation.

To date, no study has investigated the combined effect of MO and ginger on degenerative diseases. The current research investigated the effect of MO and ginger, separately, as inflammatory agents in preventing degenerative diseases. The combination of ginger and MO treatment may produce a synergistic effect in preventing degenerative disease. Hence, future studies should focus on elucidating the molecular mechanism of combined ginger and MO treatment in delaying aging and degenerative diseases.

## Figures and Tables

**Table 1:** The comparison between moringa and ginger.

Items	Moringa	Ginger	References
Scientific name	*Moringa oleifera* Lam	*Zingiber officinale* Roscoe	[21,34]
Family and genus	Moringaceae family and *Moringa* genus	Zingiberaceae family and Zingiber genus	[21,34]
Plant parts	Seed, root, leaves and flower	Rhizome	[22,35]
Bioactive compounds	Beta-carotene, quercetin, phytol, myricetin and moringine	6-gingerol, 8-gingerol, 6-shogaol, quercetin, and zingerone	[22,35]
Biological activities	Anti-inflammatory, antioxidant, hepatoprotective, antidiabetic, antiproliferative and cardioprotective activities	Anti-inflammatory, antibacterial, anticancer, antidiabetic, gastro-protective, antioxidant, and neuroprotective effects	[24,25,26,27,34,35,36]

**Table 2:** The effects of moringa on degenerative diseases from in vitro, in vivo, and human studies.

Related Disease	Constituent	Study Type	Potential Mechanism	References
Diabetes mellitus type 2	Methanolic extract of *Moringa oleifera*	In vivo study	Adverse effects caused by STZ were restored by reducing IL-6 and TNF-α levels. Reduced the activities of MD and GPx. Increased the activity of catalase.	[47]
	*Moringa oleifera* leaf	In vivo study	Reduced the levels of blood glucose, insulin, and TNF-α, and reduced follicle counts in a PCOS diabetic model.	[49]
	Aqueous leaf decoction of *Moringa oleifera*	In vivo study	Upregulated the mRNA expression of Ngn3, VEGF, IGF-1, PDX-1 and GLUT-1. Reduced the FBG and MDA levels. Improved alteration of the histopathology of liver, kidney, and pancreas tissues in diabetic rats.	[48]
	*Moringa oleifera* isothiocyanate-rich seed extract	In vivo study	Reduced body weight. Improved glucose tolerance. Reduced inflammatory gene expression. Decreased adiposity. Increased antioxidant gene expression. Inhibited gut microbiota.	[50]
	*Moringa oleifera* seed	In silico study	Improved insulin resistance. Modulated the insulin-a-related pathway and inflammatory response by acting on tyrosine-PTPN1, proto-oncogene SRC, and CASP3.	[51]
Cardiovascular disease	*Moringa oleifera* seed	In vivo study	Improved cardiac diastolic function, the appearance of pPPAR-α and δ, and plasmatic prostacyclin. Reduced fibrosis in the left ventricle and reduced left ventricular anterior wall thickness, interseptal thickness on diastole relative wall, and cardiac triglyceride level.	[58]
	*Moringa oleifera* seed	In vivo study	Reduced the levels of circulating CRP and nitrites. Decreased NF-κβ protein and iNOS expression. Reduced the level of circulating free p22^phox,^ 8-isoprostane and p47^phox^ expression. Upregulated SOD.	[60]
	*Moringa oleifera* leaf extract	In vivo study	Improved the levels of plasma insulin, SOD, CAT, GPx, and GRD. Reduction of GSH, serum glucose, TBARS, glycated haemoglobin, CD, and HP levels.	[59]
	*Moringa oleifera* seed oil	In vivo study	Reduced cardiac lactate dehydrogenase (LDH), creatinine kinase (CK,) and troponin levels. Decreased cardiac level of MDA. Increased the cardiac activity of SOD and GPx. Improved cardiac histomorphology.	[90]
	*Moringa oleifera* seed oil	In vivo study	Improvement of carbachol-induced relaxation in both mesenteric arteries and aortas. Association of mesenteric arteries with an EDHF-dependent mechanism. The aorta related to the activation of endothelial NO synthase, increment in Akt signalling and downregulation of arginase-1.	[61]
Neurodegenerative disease	*Moringa oleifera* leaf powder	In vivo study	Reduced calpain activity, Hyc-induced tau hyperphosphorylation and Aβ production. Restored levels of synaptic proteins (Synaptophysin, PSD95, Synapsin 1 and PSD93).	[64]
	*Moringa oleifera* active compound; isothiocyanate	In vitro and in vivo study	Modulated the apoptotic and inflammatory pathways and oxidative stress. Reduced the manifestation of TNF-α, TLR4, and IL-1β.	[65]
	*Moringa oleifera* leaf	In vitro study	Increased the cell viability Improved the glutathione and antioxidant enzyme levels and reduced lipid peroxidation. Inhibited mitochondrial dysfunction through regulating calcium levels and increment in mitochondrial membrane potential.	[66]
	*Moringa oleifera* seed	In vivo study	Improved cholinergic reactivity and neurogenesis in the scopolamine-induced group. Improved cholinergic system and hippocampal neurogenesis involving the Akt/ERK1/2/CREB signalling pathway.	[67]
	*Moringa oleifera* extract	In vivo study	Reduced MDA, nitrite, SOD and TNF-α. Restored cholinergic transmission by inhibiting acetylcholinesterase and maintaining the neuronal integrity in mice brains.	[68]
Cancer	*Moringa oleifera* active compound; 4-[(α-L-Rhamnosyloxy) benzyl] isothiocyanate	In vitro and in vivo study	Inhibited the growth of five types of renal cell carcinoma. Inhibited cell migration and invasion. Reduced the expression of MMP-9 and MMP-2. Induced cell cycle arrest and apoptosis. Increased the Bax/Bcl-2 ratio. Decreased protein expression. Suppressed the growth of xenograft tumours in mice and enhanced the Bax/Bcl-2 ratio in tumour tissues.	[73]
	*Moringa oleifera* fruit	In vitro study	Activation of caspase 3 enzyme and ROS-mediated apoptosis. Molecular docking analyses revealed drug-like candidates that did not exhibit any toxicity effect.	[74]
	*Moringa oleifera* leaf	In vivo study	Decreased lipid peroxidation and myeloperoxidase activity. Reduced pro-inflammatory cytokines TNF-α and IL-2.	[75]
	*Moringa oleifera* methanolic leaf extract	In vitro study	Promoted G0/G1 cell-cycle progression and apoptosis through downregulation of the Hedgehog signalling pathway. Decreased mRNA expression of the GLI1 transcription factor and SMO protein.	[76]
	*Moringa oleifera* methanolic leaf extract	In vitro study	Induced early apoptosis. Increased the expression of pro-apoptotic proteins, including Bax, caspase 8, and p53.	[77]
Kidney disease	*Moringa oleifera* leaf extract	In vivo study	Possessed high antioxidant capacity. Improved levels of CAT, SOD, GSH, GPx, and IL-6. Reduced MDA level.	[47]
	*Moringa oleifera* leaf extract	In vivo study	Reduced the levels of AST, ALT, catalase, and SOD. Increased expression of the XIAP and Bcl-2 genes. Decreased Bax and caspase 3 levels.	[82]
	*Moringa oleifera* stem extract	In vivo study	Inhibited oxidative stress and inflammation markers. Modified NF-κβ and KIM-1 signalling pathways	[83]
	*Moringa oleifera* leaf extract	In vivo study	Reduced expression of IL-6, IL-1β, TNF-α, COX2, and iNOS. Restored histopathological damage.	[84]
	*Moringa oleifera* leaf extract	In vivo study	Reduced the MDA amount, advanced protein product oxidation, and protein carbonyl, serum blood urea, and creatinine levels. Increased the activities of GPx and GST. Decreased the level of H_2_O_2_ and NO in renal tissues.	[85]

**Table 3:** The effects of ginger on degenerative diseases from in vitro, in vivo and human studies.

Related Disease	Constituent	Study Type	Potential Mechanism	References
Diabetes mellitus type 2	Ginger extract	Human study	Reduced the levels of triglycerides, fasting blood sugar, and LDL-cholesterol.	[54]
	Ginger rhizome extract	In vivo study	Ameliorated hyperlipidaemia, hyperglycaemia, and kidney function. Minimised the histological alteration in the kidney due to diabetes. Reduced the levels of IL-6 and TNF-α. Increased the levels of cytochrome c and caspase-3.	[55]
	Ginger extract	In vivo study	Reduced the level of MDA in hippocampal and cortical tissues and improved the level of CAT. Increased the levels of SOD and total thiols in cortical tissues and the hippocampus.	[56]
	Gingerol-enriched ginger	In vivo study	Improved glucose tolerance and increased the insulin content and gene expression of Claudin 3 and SOD1. Reduced the gene expression for pathways involved in mitophagy (LC3B, PINK1 and LC3B), biogenesis (TFAM and PGC-1α), mitochondrial fission (MFN1), fission (F1S1), and inflammation (NF-κβ).	[57]
Cardiovascular disease	Ginger crude extract	In vitro study	Induced the relaxation of coronary arteries. Suppressed the nitric oxide synthase and COX pathways.	[62]
	Ginger extract	In vitro study	Improved the adenosine deaminase, ADP hydrolysis, and acetylcholinesterase activities in lymphocytes of hypertensive rats. Reduced the expression of IL-10 and anti-inflammatory cytokines. Increased the levels of IL-6, IL-1, interferon-ϒ, and TNF-α. Increased serum butyrylcholinesterase activity and pro-inflammatory cytokine levels.	[43]
	Ginger extract	In vivo study	Decreased the 8-OHdG amount, β-MHC gene expression, and NADPH oxidase level. Increased significantly the level of the paraoxonase enzyme.	[63]
Neurodegenerative disease	Ginger active compound; Gingerol	In vivo study	Upregulated BDNF. Activated the protein kinase B/Akt and CREB signalling pathways.	[69]
	Ginger active compound; 6-shogaol	In vitro and in vivo study	Inhibited the Aβ aggregation in HT22 cells. Repressed the Aβ aggregation in the brain animal.	[70]
	Ginger fermented with *Schizosaccharomyces* pombe	In vivo study	Lessened memory impairment. Inhibited neuronal cell loss and synaptic disruption.	[71]
	Ginger active compound; 6-shogaol	In vitro and in vivo study	Downregulated CysLT1R. Inhibited CysLT1R/cathepsin. Reduced the Aβ deposition in the brain. Ameliorated the behavioural deficits of the AD mice model.	[72]
Cancer	Ginger active compound; 6-Gingerol	In vitro study	Repressed cell proliferation. Enhanced the sub-G1 phase ratio. Depolarised the mitochondrial membrane potential of a human bladder cell line. Downregulated BCL-2 and survivin. Upregulated Bax. Activated and regulated caspase-3, caspase-9, and MAPKs.	[78]
	Ginger active compound; 6-Shogaol	In vitro and in vivo study	Suppressed cell migration and proliferation. Cell-cycle arrest in the G_2_/M phase. Induced apoptosis via the mitochondrial pathway by downregulating the expression of p-Akt, p-mTOR, and Pl3K. Inhibited the cell proliferation and tumour growth in tumour tissues.	[79]
	Ginger active compound; 6-Shogaol	In vitro study	Improved the anticancer effect of chemotherapeutic agents particularly 5-fluorouracil, oxaliplatin, and irinotecan. Increased autophagy and apoptosis in colon cancer cells.	[80]
	Ginger active compound; 6-Shogaol	In vivo study	Reduced colon weight, length, and NO, MPO, H_2_O_2,_ and TNF-α levels. Increased expression of antioxidant enzymes SOD, CAT, GSH, and glutathione S transferase.	[81]
Kidney disease	Ginger rhizome extract	In vivo study	Reduced levels of MDA, protein carbonyl, pro-inflammatory cytokines, caspase 3, and cytochrome c. Minimised alterations in the kidney	[55]
	Ginger extract	In vivo study	Ameliorated the alteration of kidney structure. Restored biochemical changes.	[86]
	Zingerone	In vivo study	Reduced the levels of TNF-α, IL-6, and IL-1β. Decreased KIM-1, creatinine, and BUN levels. Suppressed TGF-β and LDH expression.	[87]
	Ginger extract	In vivo study	Restored TAC. Restored renal function biomarkers, histology, and molecular DNA.	[88]
	6-Gingerol	In vivo study	Restored alteration of gamma-glutamyl transferase, transaminase, ALT, LDH, bilirubin, triglyceride, cholesterol, urea, creatinine, uric acid, and BUN levels. Increased protein and albumin concentrations. Decreased lipid peroxidation. Increased the glutathione content and antioxidant enzyme activities.	[89]

## Data Availability

No new data were created in this study; all citations are publicly available.

## References

[1] Cooke J.P. (2019). Inflammation and Its Role in Regeneration and Repair. Circ. Res..

[2] Adamczyk-Sowa M., Nowak-Kiczmer M., Jaroszewicz J., Berger T. (2022). Immunosenescence and multiple sclerosis. Neurol. Neurochir. Pol..

[3] Sugimoto M.A., Vago J.P., Perretti M., Teixeira M.M. (2019). Mediators of the Resolution of the Inflammatory Response. Trends Immunol..

[4] Feehan K.T., Gilroy D.W. (2019). Is Resolution the End of Inflammation?. Trends Mol. Med..

[5] Rea I.M., Gibson D.S., McGilligan V., McNerlan S.E., Alexander H.D., Ross O.A. (2018). Age and age-related diseases: Role of inflammation triggers and cytokines. Front. Immunol..

[6] Daenen K., Andries A., Mekahli D., Van Schepdael A., Jouret F., Bammens B. (2019). Oxidative stress in chronic kidney disease. Pediatr. Nephrol..

[7] Pizzino G., Irrera N., Cucinotta M., Pallio G., Mannino F., Arcoraci V., Squadrito F., Altavilla D., Bitto A. (2017). Oxidative stress: Harms and benefits for human health. Oxidative Med. Cell. Longev..

[8] Nguyen G.T., Green E.R., Mecsas J. (2017). Neutrophils to the ROScue: Mechanisms of NADPH oxidase activation and bacterial resistance. Front. Cell. Infect. Microbiol..

[9] Hu Y.-P., Peng Y.-B., Zhang Y.-F., Wang Y., Yu W.-R., Yao M., Fu X.-J. (2017). Reactive oxygen species mediated prostaglandin E2 contributes to acute response of epithelial injury. Oxidative Med. Cell. Longev..

[10] Lilienbaum A. (2013). Relationship between the proteasomal system and autophagy. Int. J. Biochem. Mol. Biol..

[11] Park S.S., Seo Y.-K., Kwon K.-S. (2019). Sarcopenia targeting with autophagy mechanism by exercise. BMB Rep..

[12] McCormick R., Vasilaki A. (2018). Age-related changes in skeletal muscle: Changes to life-style as a therapy. Biogerontology.

[13] Hernandez-Segura A., Nehme J., Demaria M. (2018). Hallmarks of Cellular Senescence. Trends Cell Biol..

[14] Kong P., Cui Z.Y., Huang X.F., Zhang D.D., Guo R.J., Han M. (2022). Inflammation and atherosclerosis: Signaling pathways and therapeutic intervention. Signal Transduct. Target. Ther..

[15] Khandia R., Munjal A. (2020). Interplay between inflammation and cancer. Adv. Protein Chem. Struct. Biol..

[16] Rohm T.V., Meier D.T., Olefsky J.M., Donath M.Y. (2022). Inflammation in obesity, diabetes, and related disorders. Immunity.

[17] Hirano T. (2021). IL-6 in inflammation, autoimmunity and cancer. Int. Immunol..

[18] Guo J., Zheng L., Chen L., Luo N., Yang W., Qu X., Liu M., Cheng Z. (2015). Lipopolysaccharide activated TLR4/NF-κβ signaling pathway of fibroblasts from uterine fibroids. Int. J. Clin. Exp. Pathol..

[19] Moser B., Hochreiter B., Basílio J., Gleitsmann V., Panhuber A., Pardo-Garcia A., Hoesel B., Salzmann M., Resch U., Noreen M. (2021). The inflammatory kinase IKKα phosphorylates and stabilizes c-Myc and enhances its activity. Mol. Cancer.

[20] Singh S., Singh T.G. (2020). Role of nuclear factor kappa B (NF-κB) signalling in neurodegenerative diseases: An mechanistic approach. Curr. Neuropharmacol..

[21] Dhakad A.K., Ikram M., Sharma S., Khan S., Pandey V.V., Singh A. (2019). Biological, nutritional, and therapeutic significance of *Moringa oleifera* Lam. Phytother. Res..

[22] Sharma K., Kumar M., Waghmare R., Suhag R., Gupta O.P., Lorenzo J.M., Prakash S., Radha, Rais N., Sampathrajan V. (2022). Moringa (*Moringa oleifera* Lam.) polysaccharides: Extraction, characterization, bioactivities, and industrial application. Int. J. Biol. Macromol..

[23] Rodríguez-Pérez C., Quirantes-Piné R., Fernández-Gutiérrez A., Segura-Carretero A. (2015). Optimization of extraction method to obtain a phenolic compounds-rich extract from *Moringa oleifera* Lam leaves. Ind. Crops Prod..

[24] Landázuri A.C., Gualle A., Castañeda V., Morales E., Caicedo A., Orejuela-Escobar L.M. (2021). *Moringa oleifera* Lam. leaf powder antioxidant activity and cytotoxicity in human primary fibroblasts. Nat. Prod. Res..

[25] Ramamurthy S., Thiagarajan K., Varghese S., Kumar R., Karthick B.P., Varadarajan S., Balaji T.M. (2022). Assessing the In Vitro Antioxidant and Anti-inflammatory Activity of *Moringa oleifera* Crude Extract. J. Contemp. Dent. Pract..

[26] Wen Y., Liu Y., Huang Q., Liu R., Liu J., Zhang F., Liu S., Jiang Y. (2022). *Moringa oleifera* Lam. seed extract protects kidney function in rats with diabetic nephropathy by increasing GSK-3β activity and activating the Nrf2/HO-1 pathway. Phytomedicine.

[27] Alia F., Putri M., Anggraeni N., Syamsunarno M. (2021). The Potency of *Moringa oleifera* Lam. as Protective Agent in Cardiac Damage and Vascular Dysfunction. Front. Pharmacol..

[28] Ballester P., Cerdá B., Arcusa R., Marhuenda J., Yamedjeu K., Zafrilla P. (2022). Effect of Ginger on Inflammatory Diseases. Molecules.

[29] Li C., Li J., Jiang F., Tzvetkov N.T., Horbanczuk J.O., Li Y., Atanasov A.G., Wang D. (2021). Vasculoprotective effects of ginger (*Zingiber officinale* Roscoe) and underlying molecular mechanisms. Food Funct..

[30] Ebrahimzadeh A., Ebrahimzadeh A., Mirghazanfari S.M., Hazrati E., Hadi S., Milajerdi A. (2022). The effect of ginger supplementation on metabolic profiles in patients with type 2 diabetes mellitus: A systematic review and meta-analysis of randomized controlled trials. Complement. Ther. Med..

[31] Andrade C. (2021). Ginger for Migraine. J. Clin. Psychiatry.

[32] Talebi M., İlgün S., Ebrahimi V., Talebi M., Farkhondeh T., Ebrahimi H., Samarghandian S. (2021). *Zingiber officinale* ameliorates Alzheimer’s disease and Cognitive Impairments: Lessons from preclinical studies. Biomed. Pharmacother..

[33] Kiyama R. (2020). Nutritional implications of ginger: Chemistry, biological activities and signaling pathways. J. Nutr. Biochem..

[34] Mao Q.Q., Xu X.Y., Cao S.Y., Gan R.Y., Corke H., Beta T., Li H.B. (2019). Bioactive Compounds and Bioactivities of Ginger (*Zingiber officinale* Roscoe). Foods.

[35] Zhang M., Zhao R., Wang D., Wang L., Zhang Q., Wei S., Lu F., Peng W., Wu C. (2021). Ginger (*Zingiber officinale* Rosc.) and its bioactive components are potential resources for health beneficial agents. Phytother. Res..

[36] Unuofin J.O., Masuku N.P., Paimo O.K., Lebelo S.L. (2021). Ginger from Farmyard to Town: Nutritional and Pharmacological Applications. Front. Pharmacol..

[37] Liao P.-C., Lai M.-H., Hsu K.-P., Kuo Y.-H., Chen J., Tsai M.-C., Li C.-X., Yin X.-J., Jeyashoke N., Chao L.K.-P. (2018). Identification of β-sitosterol as in vitro anti-inflammatory constituent in *Moringa oleifera*. J. Agric. Food Chem..

[38] Sul O.J., Ra S.W. (2021). Quercetin Prevents LPS-Induced Oxidative Stress and Inflammation by Modulating NOX2/ROS/NF-κβ in Lung Epithelial Cells. Molecules.

[39] Abdel-Daim M.M., Khalil S.R., Awad A., Abu Zeid E.H., El-Aziz R.A., El-Serehy H.A. (2020). Ethanolic Extract of *Moringa oleifera* Leaves Influences NF-κβ Signaling Pathway to Restore Kidney Tissue from Cobalt-Mediated Oxidative Injury and Inflammation in Rats. Nutrients.

[40] Mabrok H.B., Mohamed M.S. (2019). Induction of COX-1, suppression of COX-2 and pro-inflammatory cytokines gene expression by *moringa* leaves and its aqueous extract in aspirin-induced gastric ulcer rats. Mol. Biol. Rep..

[41] Tan W.S., Arulselvan P., Karthivashan G., Fakurazi S. (2015). *Moringa oleifera* flower extract suppresses the activation of inflammatory mediators in lipopolysaccharide-stimulated RAW 264.7 macrophages via NF-κB pathway. Mediat. Inflamm..

[42] Ezzat S.M., Ezzat M.I., Okba M.M., Menze E.T., Abdel-Naim A.B. (2018). The hidden mechanism beyond ginger (*Zingiber officinale* Rosc.) potent in vivo and in vitro anti-inflammatory activity. J. Ethnopharmacol..

[43] Akinyemi A.J., Thomé G.R., Morsch V.M., Bottari N.B., Baldissarelli J., de Oliveira L.S., Goularte J.F., Bello-Klein A., Duarte T., Duarte M. (2016). Effect of ginger and turmeric rhizomes on inflammatory cytokines levels and enzyme activities of cholinergic and purinergic systems in hypertensive rats. Planta Medica.

[44] Kim Y., Kim D.M., Kim J.Y. (2017). Ginger extract suppresses inflammatory response and maintains barrier function in human colonic epithelial Caco-2 cells exposed to inflammatory mediators. J. Food Sci..

[45] Hamza A.A., Heeba G.H., Hamza S., Abdalla A., Amin A. (2021). Standardized extract of ginger ameliorates liver cancer by reducing proliferation and inducing apoptosis through inhibition oxidative stress/ inflammation pathway. Biomed. Pharmacother..

[46] Villarruel-López A., López-de la Mora D.A., Vázquez-Paulino O.D., Puebla-Mora A.G., Torres-Vitela M.R., Guerrero-Quiroz L.A., Nuño K. (2018). Effect of *Moringa oleifera* consumption on diabetic rats. BMC Complement. Altern. Med..

[47] Omodanisi E.I., Aboua Y.G., Oguntibeju O.O. (2017). Assessment of the anti-hyperglycaemic, anti-inflammatory and antioxidant activities of the methanol extract of *Moringa oleifera* in diabetes-induced nephrotoxic male wistar rats. Molecules.

[48] Kamaliani B., Setiasih N., Winaya I. (2019). Histopathological kidney overview of experimental diabetes mellitus Wistar rats given ethanol extract of Moringa leaf. Bul. Vet. Udayana.

[49] Siahaan S., Santoso B., Widjiati (2022). Effectiveness of *Moringa oleifera* Leaves on TNF-α Expression, Insulin Levels, Glucose Levels and Follicle Count in Rattus norvegicus PCOS Model. Diabetes Metab. Syndr. Obes..

[50] Jaja-Chimedza A., Zhang L., Wolff K., Graf B.L., Kuhn P., Moskal K., Carmouche R., Newman S., Salbaum J.M., Raskin I. (2018). A dietary isothiocyanate-enriched moringa (*Moringa oleifera*) seed extract improves glucose tolerance in a high-fat-diet mouse model and modulates the gut microbiome. J. Funct. Foods.

[51] Huang Q., Liu R., Liu J., Huang Q., Liu S., Jiang Y. (2020). Integrated Network Pharmacology Analysis and Experimental Validation to Reveal the Mechanism of Anti-Insulin Resistance Effects of *Moringa oleifera* Seeds. Drug Des. Dev. Ther..

[52] Veisi P., Zarezade M., Rostamkhani H., Ghoreishi Z. (2022). Renoprotective effects of the ginger (*Zingiber officinale*) on Diabetic kidney disease, current knowledge and future direction: A systematic review of animal studies. BMC Complement. Med. Ther..

[53] Rostamkhani H., Veisi P., Niknafs B., Jafarabadi M.A., Ghoreishi Z. (2023). The effect of *Zingiber officinale* on prooxidant-antioxidant balance and glycemic control in diabetic patients with ESRD undergoing hemodialysis: A double-blind randomized control trial. BMC Complement. Med. Ther..

[54] Carvalho G.C.N., Lira-Neto J.C.G., Araújo M.F.M., Freitas R., Zanetti M.L., Damasceno M.M.C. (2020). Effectiveness of ginger in reducing metabolic levels in people with diabetes: A randomized clinical trial. Rev. Lat. Am. Enferm..

[55] Al Hroob A.M., Abukhalil M.H., Alghonmeen R.D., Mahmoud A.M. (2018). Ginger alleviates hyperglycemia-induced oxidative stress, inflammation and apoptosis and protects rats against diabetic nephropathy. Biomed. Pharmacother..

[56] Marefati N., Abdi T., Beheshti F., Vafaee F., Mahmoudabady M., Hosseini M. (2021). *Zingiber officinale* (Ginger) hydroalcoholic extract improved avoidance memory in rat model of streptozotocin-induced diabetes by regulating brain oxidative stress. Horm. Mol. Biol. Clin. Investig..

[57] Wang R., Santos J.M., Dufour J.M., Stephens E.R., Miranda J.M., Washburn R.L., Hibler T., Kaur G., Lin D., Shen C.L. (2022). Ginger Root Extract Improves GI Health in Diabetic Rats by Improving Intestinal Integrity and Mitochondrial Function. Nutrients.

[58] Randriamboavonjy J.I., Loirand G., Vaillant N., Lauzier B., Derbré S., Michalet S., Pacaud P., Tesse A. (2016). Cardiac protective effects of *Moringa oleifera* seeds in spontaneous hypertensive rats. Am. J. Hypertens..

[59] Aju B.Y., Rajalakshmi R., Mini S. (2019). Protective role of *Moringa oleifera* leaf extract on cardiac antioxidant status and lipid peroxidation in streptozotocin induced diabetic rats. Heliyon.

[60] Randriamboavonjy J.I., Rio M., Pacaud P., Loirand G., Tesse A. (2017). *Moringa oleifera* seeds attenuate vascular oxidative and nitrosative stresses in spontaneously hypertensive rats. Oxidative Med. Cell. Longev..

[61] Randriamboavonjy J.I., Heurtebise S., Pacaud P., Loirand G., Tesse A. (2019). *Moringa oleifera* seeds improve aging-related endothelial dysfunction in wistar rats. Oxidative Med. Cell. Longev..

[62] Wu H.C., Horng C.T., Tsai S.C., Lee Y.L., Hsu S.C., Tsai Y.J., Tsai F.J., Chiang J.H., Kuo D.H., Yang J.S. (2018). Relaxant and vasoprotective effects of ginger extracts on porcine coronary arteries. Int. J. Mol. Med..

[63] Shirpoor A., Zerehpoosh M., Ansari M.H.K., Kheradmand F., Rasmi Y. (2017). Ginger extract mitigates ethanol-induced changes of alpha and beta—Myosin heavy chain isoforms gene expression and oxidative stress in the heart of male wistar rats. DNA Repair.

[64] Mahaman Y.A.R., Huang F., Wu M., Wang Y., Wei Z., Bao J., Salissou M.T.M., Ke D., Wang Q., Liu R. (2018). *Moringa oleifera* Alleviates Homocysteine-Induced Alzheimer’s Disease-Like Pathology and Cognitive Impairments. J. Alzheimers Dis..

[65] Giacoppo S., Rajan T.S., De Nicola G.R., Iori R., Rollin P., Bramanti P., Mazzon E. (2017). The isothiocyanate isolated from *Moringa oleifera* shows potent anti-inflammatory activity in the treatment of murine subacute Parkinson’s disease. Rejuvenation Res..

[66] González-Burgos E., Ureña-Vacas I., Sánchez M., Gómez-Serranillos M.P. (2021). Nutritional value of *Moringa oleifera* Lam. leaf powder extracts and their neuroprotective effects via antioxidative and mitochondrial regulation. Nutrients.

[67] Zhou J., Yang W.-s., Suo D.-q., Li Y., Peng L., Xu L.-x., Zeng K.-y., Ren T., Wang Y., Zhou Y. (2018). *Moringa oleifera* Seed Extract Alleviates Scopolamine-Induced Learning and Memory Impairment in Mice. Front. Pharmacol..

[68] Onasanwo S.A., Adamaigbo V.O., Adebayo O.G., Eleazer S.E. (2021). *Moringa oleifera*-supplemented diet protect against cortico-hippocampal neuronal degeneration in scopolamine-induced spatial memory deficit in mice: Role of oxido-inflammatory and cholinergic neurotransmission pathway. Metab. Brain Dis..

[69] Kim C.Y., Seo Y., Lee C., Park G.H., Jang J.H. (2018). Neuroprotective Effect and Molecular Mechanism of [6]-Gingerol against Scopolamine-Induced Amnesia in C57BL/6 Mice. Evid. Based Complement. Altern. Med..

[70] Na J.-Y., Song K., Lee J.-W., Kim S., Kwon J. (2017). Sortilin-related receptor 1 interacts with amyloid precursor protein and is activated by 6-shogaol, leading to inhibition of the amyloidogenic pathway. Biochem. Biophys. Res. Commun..

[71] Huh E., Lim S., Kim H.G., Ha S.K., Park H.-Y., Huh Y., Oh M.S. (2018). Ginger fermented with Schizosaccharomyces pombe alleviates memory impairment via protecting hippocampal neuronal cells in amyloid beta 1–42 plaque injected mice. Food Funct..

[72] Na J.Y., Song K., Lee J.W., Kim S., Kwon J. (2016). 6-Shogaol has anti-amyloidogenic activity and ameliorates Alzheimer’s disease via CysLT1R-mediated inhibition of cathepsin B. Biochem. Biophys. Res. Commun..

[73] Xie J., Qian Y.Y., Yang Y., Peng L.J., Mao J.Y., Yang M.R., Tian Y., Sheng J. (2021). Isothiocyanate From *Moringa oleifera* Seeds Inhibits the Growth and Migration of Renal Cancer Cells by Regulating the PTP1B-dependent Src/Ras/Raf/ERK Signaling Pathway. Front. Cell Dev. Biol..

[74] Siddiqui S., Upadhyay S., Ahmad I., Hussain A., Ahamed M. (2021). Cytotoxicity of *Moringa oleifera* fruits on human liver cancer and molecular docking analysis of bioactive constituents against caspase-3 enzyme. J. Food Biochem..

[75] Cuellar-Núñez M.L., Gonzalez de Mejia E., Loarca-Piña G. (2021). *Moringa oleifera* leaves alleviated inflammation through downregulation of IL-2, IL-6, and TNF-α in a colitis-associated colorectal cancer model. Food Res. Int..

[76] Khan F., Pandey P., Ahmad V., Upadhyay T.K. (2020). *Moringa oleifera* methanolic leaves extract induces apoptosis and G0/G1 cell cycle arrest via downregulation of Hedgehog Signaling Pathway in human prostate PC-3 cancer cells. J. Food Biochem..

[77] Mohd Fisall U.F., Ismail N.Z., Adebayo I.A., Arsad H. (2021). Dichloromethane fraction of *Moringa oleifera* leaf methanolic extract selectively inhibits breast cancer cells (MCF7) by induction of apoptosis via upregulation of Bax, p53 and caspase 8 expressions. Mol. Biol. Rep..

[78] Choi N.R., Choi W.G., Kwon M.J., Woo J.H., Kim B.J. (2022). [6]-Gingerol induces Caspase-Dependent Apoptosis in Bladder Cancer cells via MAPK and ROS Signaling. Int. J. Med. Sci..

[79] Pei X.D., He Z.L., Yao H.L., Xiao J.S., Li L., Gu J.Z., Shi P.Z., Wang J.H., Jiang L.H. (2021). 6-Shogaol from ginger shows anti-tumor effect in cervical carcinoma via PI3K/Akt/mTOR pathway. Eur. J. Nutr..

[80] Woźniak M., Makuch S., Winograd K., Wiśniewski J., Ziółkowski P., Agrawal S. (2020). 6-Shogaol enhances the anticancer effect of 5-fluorouracil, oxaliplatin, and irinotecan via increase of apoptosis and autophagy in colon cancer cells in hypoxic/aglycemic conditions. BMC Complement. Med. Ther..

[81] Ajeigbe O.F., Maruf O.R., Anyebe D.A., Opafunso I.T., Ajayi B.O., Farombi E.O. (2022). 6- shogaol suppresses AOM/DSS-mediated colorectal adenoma through its antioxidant and anti-inflammatory effects in mice. J. Food Biochem..

[82] Soliman M.M., Aldhahrani A., Alkhedaide A.Q., Nassan M.A., Althobaiti F., Mohamed W.A. (2020). The ameliorative impacts of *Moringa oleifera* leaf extract against oxidative stress and methotrexate-induced hepato-renal dysfunction. Biomed. Pharmacother..

[83] Adedapo A.A., Etim U., Falayi O.O., Ogunpolu B.S., Omobowale T.O., Oyagbemi A.A., Oguntibeju O.O. (2020). Methanol stem extract of *Moringa oleifera* mitigates glycerol-induced acute kidney damage in rats through modulation of KIM-1 and NF-κβ signaling pathways. Sci. Afr..

[84] Tang Y., Choi E.J., Han W.C., Oh M., Kim J., Hwang J.Y., Park P.J., Moon S.H., Kim Y.S., Kim E.K. (2017). *Moringa oleifera* from Cambodia Ameliorates Oxidative Stress, Hyperglycemia, and Kidney Dysfunction in Type 2 Diabetic Mice. J. Med. Food.

[85] Akinrinde A.S., Oduwole O., Akinrinmade F.J., Bolaji-Alabi F.B. (2020). Nephroprotective effect of methanol extract of *Moringa oleifera* leaves on acute kidney injury induced by ischemia-reperfusion in rats. Afr. Health Sci..

[86] Shirpoor A., Rezaei F., Fard A.A., Afshari A.T., Gharalari F.H., Rasmi Y. (2016). Ginger extract protects rat’s kidneys against oxidative damage after chronic ethanol administration. Biomed. Pharmacother..

[87] Rehman M.U., Rashid S.M., Rasool S., Shakeel S., Ahmad B., Ahmad S.B., Madkhali H., Ganaie M.A., Majid S., Bhat S.A. (2019). Zingerone (4-(4-hydroxy-3-methylphenyl) butan-2-one) ameliorates renal function via controlling oxidative burst and inflammation in experimental diabetic nephropathy. Arch. Physiol. Biochem..

[88] Gabr S.A., Alghadir A.H., Ghoniem G.A. (2019). Biological activities of ginger against cadmium-induced renal toxicity. Saudi J. Biol. Sci..

[89] Joshi D., Srivastav S.K., Belemkar S., Dixit V.A. (2017). *Zingiber officinale* and 6-gingerol alleviate liver and kidney dysfunctions and oxidative stress induced by mercuric chloride in male rats: A protective approach. Biomed. Pharmacother..

[90] Saka W.A., Ayoade T.E., Akhigbe T.M., Akhigbe R.E. (2021). *Moringa oleifera* seed oil partially abrogates 2,3-dichlorovinyl dimethyl phosphate (Dichlorvos)-induced cardiac injury in rats: Evidence for the role of oxidative stress. J. Basic Clin. Physiol. Pharmacol..

